# NMR probe of suppressed bulk conductivity in the topological insulator Bi_0.5_Sb_1.5_Te_3_

**DOI:** 10.1039/d1ra07194g

**Published:** 2022-01-19

**Authors:** Jun Kue Park, Do Hoon Kang, Sung Kyun Park, Jae Sang Lee

**Affiliations:** Korea Multi-purpose Accelerator Complex, Korea Atomic Energy Research Institute Gyeongju 38180 Korea jkuepark@kaeri.re.kr

## Abstract

The relaxation behavior in the topological insulator (TI) Bi_0.5_Sb_1.5_Te_3_ has been investigated using ^125^Te nuclear magnetic resonance spectroscopy. We systematically investigate the spin–lattice relaxation rate (1/*T*_1_) in bulk electronic states with varying particle sizes. By analyzing the 1/*T*_1_ relaxation behavior, we find that with decreasing particle sizes the electronic states in the bulk exhibit more topological insulating behavior, indicative of an increasing energy gap supported by higher thermal activation energy. Besides, the decreasing density of states at the Fermi level was observed in the massive Dirac electrons with decreasing particle size by analyzing the spin–lattice relaxation according to a theoretical model in this spin–orbit coupled system.

## Abstract 2

We investigated insulating behaviors in the bulk of the topological insulator Bi_0.5_Sb_1.5_Te_3_ varying in particle size using ^125^Te NMR spectroscopy, within the framework of a theoretical relaxation model of the Dirac electron system.

## Graphical abstract

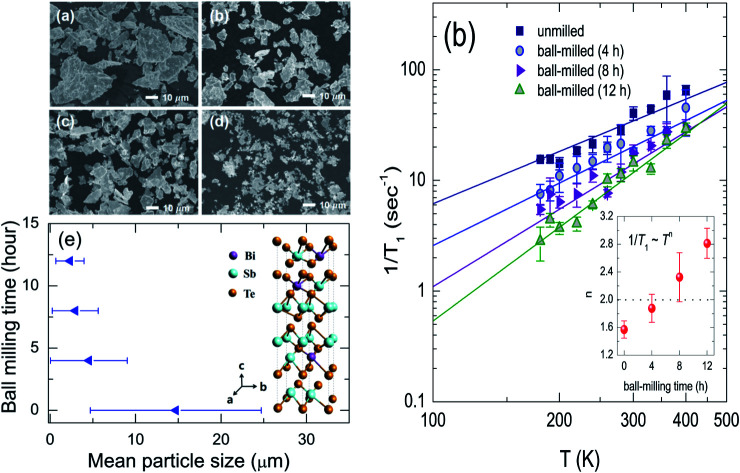

## 1. Introduction

Topological insulators (TIs) of layered materials with binary or ternary chalcogenides have been widely investigated due to the interest in potential applications such as spintronics and quantum computing.^1–3^ An ideal TI exhibits gapless Dirac-like edge or surface states, whereas a bulk interior is featured by an insulating energy gap.^1,2,4,5^ The suppressed conductivity of the bulk interior in TIs is important as much as the edge or surface states given the applications such as field-effect transistors, because the insulating bulk state may act in a role of switch off, but the topological conductive state acts as switch on. Here, we demonstrate an improved topological insulating state in the bulk by decreasing particle sizes of the TI Bi_0.5_Sb_1.5_Te_3_.

Some previous studies reported the surface and bulk electronic states using nuclear magnetic resonance (NMR) spectroscopy by varying the particle sizes. NMR typically probes the bulk properties, and relaxation from the surface hardly contributes.^6,7^ Thus, in micrometer-sized TIs, the NMR spectroscopy may be likely to probe the bulk electronic states regarding carrier concentration, thermal activation energy, and density of states at the Fermi level. In a nanoscale TI, however, Koumoulis *et al.*^6^ found the metallic surface state obeying the Korringa relation, with a well-separated NMR shoulder peak arising from a greater surface-to-volume ratio. In this metallic state, the spin–lattice relaxation rate (1/*T*_1_) is determined by the interaction between the nucleus and electrons of the surface topological state.

Recently, a theoretical model of the spin–lattice relaxation in NMR has been developed for this spin–orbit coupled system.^8–12^ The Dirac electron gas in the three-dimensional bulk system has the general property of large diamagnetism.^13–15^ Intriguingly, when the Fermi level is located in the bandgap, the magnitude of diamagnetism reaches a maximum.^14,15^ The interband effect of the magnetic field solves the unexpected large diamagnetism of small gap insulators, which cannot be described by the standard theory of diamagnetism, *i.e.*, the Landau–Peierls formula.^10,14,15^ This effect induces large diamagnetism as well as a significant enhancement in the permittivity, giving rise to a large Knight shift, and it is a general property of Dirac electron systems. In this work, we systematically study the Dirac electron system in the bulk of a TI with varying particle sizes, according to the recent theoretical model.^10^ We observed a varying carrier density and obtained activation energies by analyzing the 1/*T*_1_ data for the TI Bi_0.5_Sb_1.5_Te_3_.

## 2. Experimental

Polycrystalline Bi_0.5_Sb_1.5_Te_3_ with a purity of 99.99% from Sigma Aldrich was used as received without further purification. The as-received powder consisted of a large (14.7 ± 10.0 μm) average grain size, which was reduced to various smaller particle sizes by ball milling under inert atmosphere. The particle sizes measured are mean values determined with a field emission scanning electron microscope (SEM). Powder X-ray diffraction (PXRD) was also used to confirm the crystallinity of the samples using a Philips XPERT MPD X-ray diffractometer with Cu Kα (*λ* = 1.5405 Å) radiation. ^125^Te NMR data were acquired with a Bruker Avance II^+^-400 solid-state NMR spectrometer operating at 126.3 MHz. Spectral data were acquired using a spin-echo sequence [(π/2)_*x*_ − *τ* − (π)_*y*_-acquire] with the echo delay *τ* set to 20 μs. The NMR spectra were obtained at various temperatures of 180 K to 400 K. Spin–lattice relaxation time (*T*_1_) data were acquired by the saturation-recovery technique and fitted to the stretched exponential function.^7^ The ^125^Te NMR chemical shift was externally referenced to a solution of telluric acid, Te(OH)_6_, calibrated at 692.2 ppm.

## 3. Results and discussion

### 3.1 Crystal structure analysis of the Bi_0.5_Sb_1.5_Te_3_ particles

**Fig. 1:**
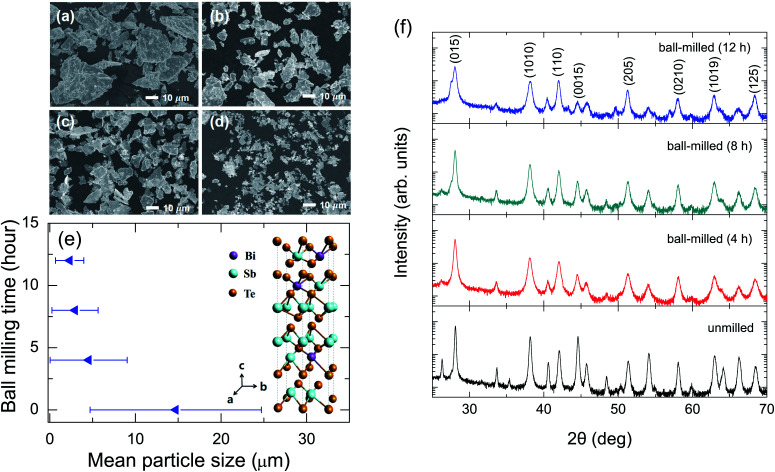
SEM images of (a) unmilled and ball-milled samples for (b) 4 h, (c) 8 h, and (d) 12 h (upper left panel). (e) Particle size distribution with varying ball-milling times. The mean particle sizes and their deviations were 14.7 ± 10.0 μm, 4.5 ± 4.5 μm, 2.9 ± 2.7 μm, and 2.3 ± 1.7 μm for unmilled and ball-milled for 4 h, 8 h, and 12 h, respectively, obtained from the SEM images. Inset of (e) shows crystal structure of Bi_0.5_Sb_1.5_Te_3_. (f) PXRD patterns of the unmilled and ball-milled samples for 4 h, 8 h, and 12 h.

Fig. 1(a)–(d) show scanning electron microscope (SEM) images for the samples before and after the ball milling. With increasing the ball-milling time, the mean size of the particles decreases as expected, and the particle size distribution shows a decrease as shown in Fig. 1(e), indicating the inhomogeneity of the particle sizes is decreased by the ball milling. For the ball-milled (12 h) sample [Fig. 1(d)], the formation of clusters in the particles is more obvious compared with the other samples. In inset of Fig. 1(e), the crystal structure of Bi_0.5_Sb_1.5_Te_3_ consisting of quintuple layers along the *c*-axis is displayed. The quintuple layers are weakly coupled with each other by van der Waals interactions.^16,17^Fig. 1(f) shows the powder X-ray diffraction (PXRD) data for the samples before and after the ball milling. All the diffraction peaks can be indexed based on the standard JCPDS #49-1713 of Bi_0.5_Sb_1.5_Te_3_.^16,18,19^ The diffraction peak at approximately 27.6° denoted by asterisks may be attributed to (101) planes of elementary Te, indicative of an introduced second-phase peak after performing the ball-milling more than 4 h.^19,20^ With increasing the ball-milling time, the XRD peaks exhibit the line broadening indicative of decreasing crystallite sizes according to the Debye–Scherrer formula.^4,21^

### 3.2 NMR line shapes and relaxation patterns

**Fig. 2:**
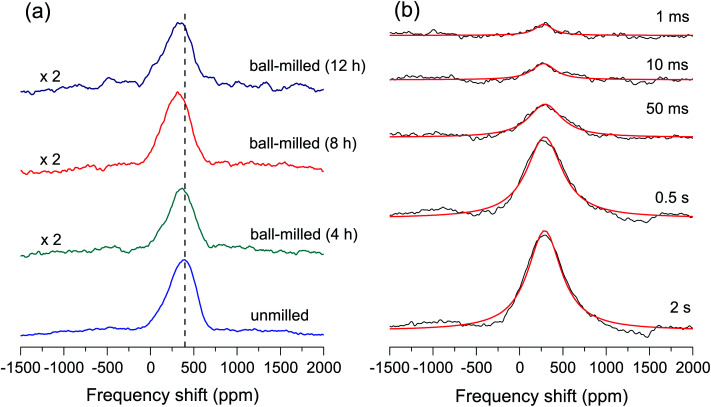
(a) ^125^Te NMR spectra measured at 400 K for the unmilled and ball-milled samples. All the spectra were well fitted to a single Lorentzian function. The linewidths obtained were 346 ± 2 ppm, 349 ± 2 ppm, 367 ± 2 ppm, and 381 ± 2 ppm for the unmilled and ball-milled for 4 h, 8 h, and 12 h, respectively. A vertical dashed line denotes a peak frequency of the unmilled sample. (b) ^125^Te NMR spectra when acquired during the saturation recovery relaxation experiments at different delay times at 190 K for a sample ball-milled for 12 h.

Fig. 2(a) shows ^125^Te NMR spectra of the samples before and after the ball milling. With decreasing particle size, the peak shifts toward lower frequencies, and the spectra show a line broadening with increasing increments up to the ball-milled sample for 8 h, after which with a decreasing increment for 12 h. The frequency shift can be separated into a spin and an orbital contributions, *K* = *K*_spin_ + *K*_orbital_.^22,23^ A larger orbital diamagnetism may affect a remarkable NMR shift in a system with a strong spin–orbit coupling.^10,12,24^ A remarkable NMR shift was previously observed, when a topological band inversion dictating a topological phase transition in the bulk takes place^12^ or the Fermi level is on the Dirac semimetal state in the bandgap, but those are not our case.^9,10,12,25,26^ Previously, the topological metallic surface state may exhibit a negative NMR shift (Δ*ν* ∼ 500 ppm) and line broadening (Δ*H* ∼ 400 ppm) caused by the spin scattering from the metallic surface states with decreasing particle sizes in nanoscale TIs Bi_2_Te_3_.^6^ Compared with the previous results, a slight shift (Δ*ν* = 44 ppm) and a line broadening (Δ*H* = 35 ppm) on our micrometer-sized TIs, thus, may imply the bulk properties [see Fig. 2(a)]. We now discuss how the slight frequency shifts and the line broadening arising from the bulk take place upon decreasing the sizes of the particles. The ball-milling processes may introduce a local structural disorder in the particles as revealed by XRD data [Fig. 1(f)], which may cause the NMR frequency shifts and the line broadening leading to distortions in the local magnetic field. Previously, Sn doping in a topological crystalline insulator PbTe may cause structural inhomogeneity, giving NMR shifts and a line broadening in agreement with our results.^5^

**Fig. 3:**
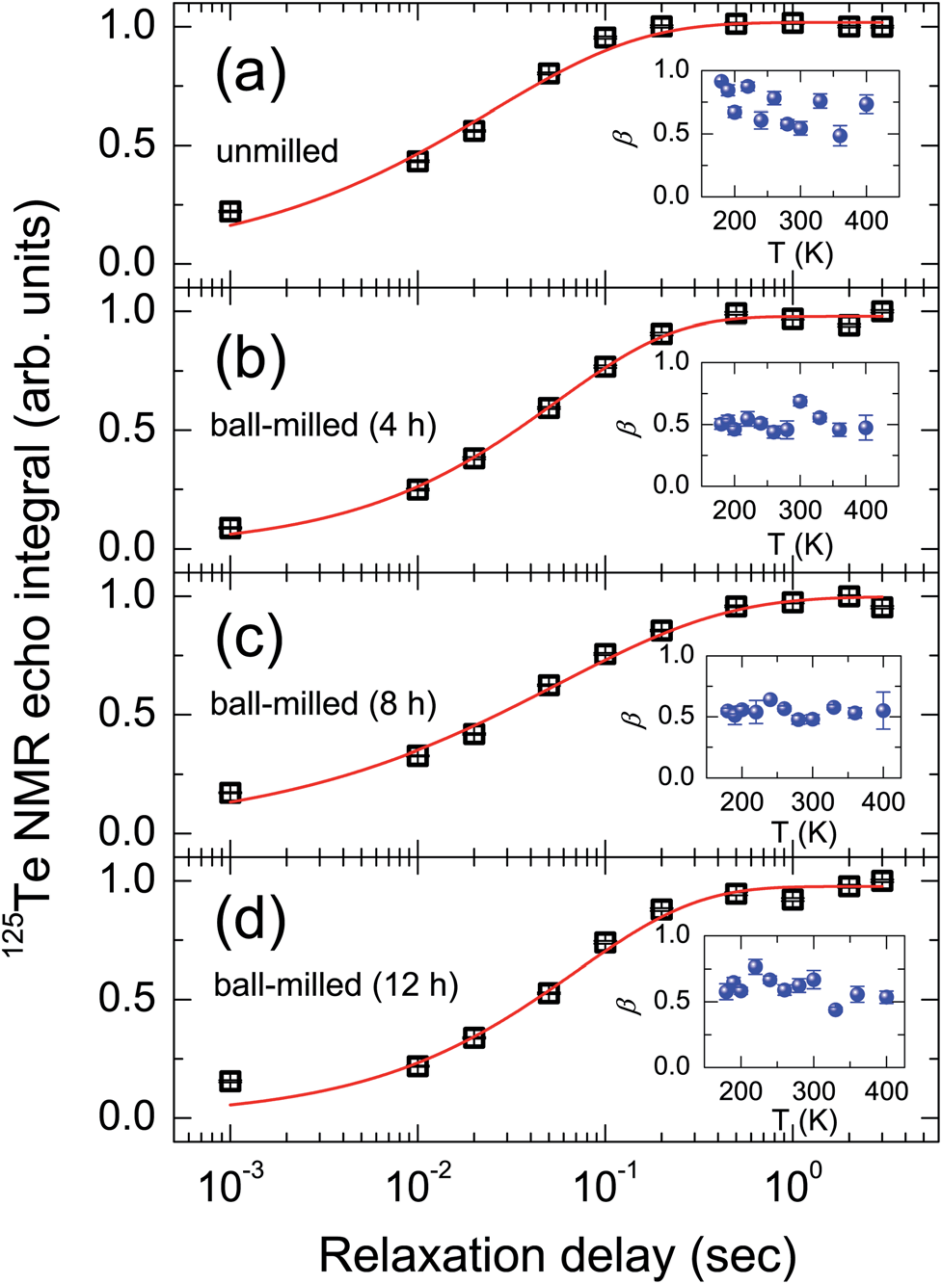
^125^Te NMR magnetization recovery curves acquired at 300 K for (a) unmilled and ball-milled samples for (b) 4 h, (c) 8 h, and (d) 12 h. The solid red line denotes the stretched exponential fit (see text). Insets of the figure show the exponent *β* obtained by the fits as a function of temperature.

Fig. 2(b) shows the spectra acquired at different delay times during the experiments of the saturation recovery relaxation for the ball-milled sample for 12 h. All the spectra exhibiting some asymmetric line shape were fitted to a single Lorentzian function. Fig. 3 exhibits the intensities obtained from a single Lorentzian fit to the spectra at 300 K as a function of relaxation delay for each sample. The spin–lattice relaxation measured by the saturation-recovery could be well fitted to a stretched exponential function,^4,6,27–29^

1 *M*(*t*) = *M*_0_[1 − exp(−(*t*/*T*_1_)^*β*^)],

where *β* is the stretching parameter (0 < *β* < 1). The fit with the stretched exponential function indicates an extended distribution of *T*_1_ times caused by the different local environments of Te sites, as well as by the local distribution. The asymmetric line shape in the spectra also indicates the inhomogeneous local environments.^7^

### 3.3 Relaxation behavior of particle-sized topological insulators

**Fig. 4:**
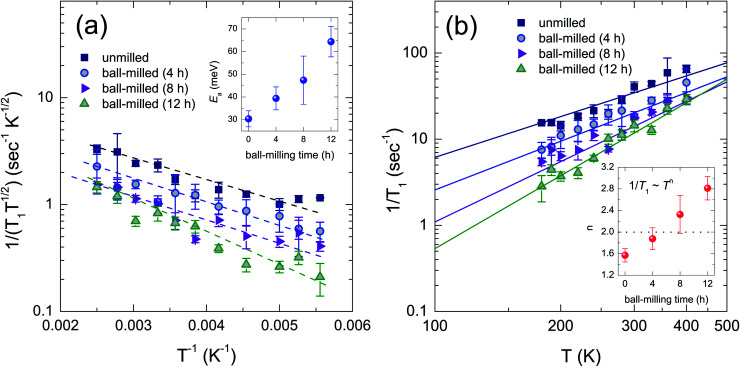
(a) A semi-logarithmic plot of 1/(*T*_1_*T*^0.5^) as a function of inverse temperature for all the samples before and after ball milling. The dashed lines show a linear behavior of the plot as a guide to the eyes. Inset of (a) shows the activation energy obtained by a fit with an Arrhenius law to 1/*T*_1_ data. (b) A semi-logarithmic plot of 1/*T*_1_ as a function of temperature for all the samples. The solid lines represent a fit to the data with a power-law *T*^*n*^, where the exponent *n* was 1.57 ± 0.12, 1.88 ± 0.20, 2.33 ± 0.35, and 2.81 ± 0.22 for the unmilled and the ball-milled samples for 4 h, 8 h, and 12 h, respectively [inset of (b)].

Fig. 4(a) shows the 1/(*T*_1_*T*^0.5^) as a function of inverse temperature for all the samples. The quantity 1/(*T*_1_*T*^0.5^) is proportional to the carrier concentration (*N*) in a semiconductor,^6,30^ featuring a more topological insulating behavior with decreasing particle sizes across the entire temperature range. We note that it is unlikely to observe the surface electronic states due to their micrometer-sized particles even by the longest ball-milling time in our samples. In Fig. 4(b), we plot 1/*T*_1_ for the unmilled and ball-milled samples as a function of temperature. It appears that 1/*T*_1_ data for each sample show a distinct temperature dependence across the entire temperature range.

The 1/*T*_1_ data were well fitted to a power-law function of temperature (∼*T*^*n*^),^4,5,9,10^ where the extracted fitting parameter *n* is displayed in inset of Fig. 4. When the 1/*T*_1_ exhibits *T*^*n*≥2^ dependence, the two-phonon Raman relaxation process is predominant.^4^ The inelastic scatting of phonons by the spin may contribute the relaxation in the ball-milled samples for 8 h and 12 h. On the other hand, the 1/*T*_1_ ∝ *T*^*n*<2^ implies a slow temperature dependence. For the samples of unmilled and ball-milled for 4 h, the relaxation process may dominantly arise from the interaction with the conductive electrons rather than the Raman process.^4,31^ Moreover, the power-law behavior of 1/*T*_1_ depending on the temperature may be interpreted based on a theoretical model of the Dirac electron system.^10^ According to the theory, when the system goes to the massless Dirac electron from the massive Dirac electron, 1/*T*_1_ is proportional to *T*^3^ dependence from *T* linear, implying zero density of state at the Fermi level. Hence, with decreasing particle sizes, the increasing slope of 1/*T*_1_ data in Fig. 4 may lead to decreasing density of state at the Fermi level in this system.

We further analyzed the 1/*T*_1_ data with an Arrhenius law to obtain activation energy in this system.^4,31^ By fitting to the data, we calculated the energies of 30.4 ± 3.5, 39.4 ± 5.0, 47.4 ± 10.6, and 64.4 ± 6.7 meV for the unmilled and ball-milled samples for 4 h, 8 h, and 12 h, respectively [see inset of Fig. 4(a)]. As the particle size decreases, greater activation energy was obtained, indicating suppressed bulk conductivities of this system. We thus suggest that the topological insulating bandgap of the bulk becomes greater with decreasing particle size as we have observed from 1/*T*_1_ behavior. Comparing our 1/*T*_1_ behavior with that of previous micrometer-sized Bi_0.5_Sb_1.5_Te_3_,^4^ Koumoulis *et al.* obtained comparable activation energy of 51 meV and a linear trend across the same temperature range as ours. The linear trend may be attributed to more metallic properties of this sample possibly due to the introduction of the element Sb.^4^ As a result, our finding suggests that the bulk electronic property of Bi_0.5_Sb_1.5_Te_3_ becomes more insulating states with decreasing particle sizes. The ball-milling process yielding smaller particles may increase structural disorders both on the surface and the bulk of this TI.

## 4. Conclusions

In summary, we have investigated the relaxation behavior in a Dirac electron system of Bi_0.5_Sb_1.5_Te_3_ by employing the NMR spectroscopy. With increasing ball-milling time, the particle size and the inhomogeneity in the sizes decreased. Negligible frequency shifts and the change in the linewidths of the NMR spectra with decreasing particle sizes imply the bulk electronic properties of the TI. We observed topological insulating behavior in the bulk with decreasing particle sizes due to increasing slopes in 1/*T*_1_ explained by two-phonon Raman relaxation and the increasing activation energy obtained by an Arrhenius fit to the 1/*T*_1_ data. Moreover, based on the theoretical model of the Dirac electron, the increasing slopes in 1/*T*_1_ may lead to decreasing density of state at the Fermi level in this system with decreasing particle sizes. We thus suggest that the insulating behavior in the bulk may arise from decreasing particle sizes and decreasing inhomogeneity in the sizes.

## Author contributions

J. K. Park and J. S. Lee contributed to the conception and design of the work. D. H. Kang and J. K. Park planned and designed the experiments, and made data acquisition. D. H. Kang prepared various sized particles by performing the ball milling. S. K. Park and J. K. Park contributed to analyze and interpret the NMR data. J. K. Park, D. H. Kang, and J. S. Lee contributed to the analysis, data interpretation characterizing the samples. All authors have reviewed and approved to the final version of the manuscript. J. K. Park supervised the project.

## Conflicts of interest

There are no conflicts to declare.

## Supplementary Material
